# The complete chloroplast genome of *Malva wigandii* (Alef.) M.F. Ray (Malvaceae, Malvoideae)

**DOI:** 10.1080/23802359.2021.1902409

**Published:** 2021-03-24

**Authors:** Lluís García-Mir, Darío I. Ojeda, Javier Fuertes-Aguilar

**Affiliations:** a Real Jardín Botánico, RJB-CSIC, Madrid, Spain; b NIBIO, Ås, Norway

**Keywords:** NGS, plastome, polyploidy, *Lavatera maritima*

## Abstract

The complete chloroplast genome sequence of wild sea mallow *Malva wigandii* (=*Lavatera maritima*) was determined and characterized in this study. The genome is 158,162 bp long, containing a pair of inverted repeats regions (IRs) of 25,166 bp, which are separated by a large single-copy region of 86,860 bp and a small single-copy region of 20,970 bp. The sea mallow chloroplast genome has 131 known genes, including 85 protein-coding genes, eight ribosomal *RNA* genes, and 37 *tRNA* genes. The phylogenomic analysis showed that *M. wigandii* forms a cluster with *Althaea officinalis* with a strong bootstrap support and is sister to sequences belonging to the tribe Gossypieae. All of them are grouped in a lineage with other members of the subfamily Malvoideae. This newly sequenced chloroplast genome sequence provides useful genetic information to explore the origin and evolution of the Mediterranean radiation that gave rise to the generic alliance of *Malva*.

The generic alliance of *Malva* encompasses four genera and approximately 60 species grouped in a clade belonging in the tribe Malveae (Malvacae). The alliance has its center of diversity in the Mediterranean region and in some invasive weeds – e.g. *Malva parviflora*, *Malva neglecta-*, found in crops from temperate regions around the world. One of the main genomic signatures of this group of species is the frequent occurrence of polyploidy, with species ranging from diploid to 16x level of ploidy (Escobar García et al. 2009). The study of the chloroplast genomes and their interactions with the nuclear genome is of particular interest specially to determine the origin of allopolyploids present in the genus *Malva*.

*Malva wigandii* (Alef.) M.F. Ray (=*Lavatera maritima* Gouan) is one of the earliest-diverging species within the *Malva* lineage, exhibiting both a broad climatic niche and a preference for basic, phosphate-rich soils. This makes it a suitable model to study the origin of invasive traits observed in other closely related species of the genus. Here, we present the complete chloroplast genome of *Malva wigandii*, a species belonging to the generic alliance of *Malva* living on limestone rocky soils of E Mediterranean region and NE Africa (Villa-Machío et al. 2018).

Leaf samples were sampled from the living collection at the Real Jardín Botánico-CSIC, Madrid, Spain (40°24′36.2′′N 3°41′20.1′′W) (accession #04803.19). Total genomic DNA was extracted with QIAGEN DNeasy Plant Mini Kit (QIAGEN, Manchester, UK). Two pair-ended 150-bp libraries were prepared with TruSeq DNA PCR-Free kit (Illumina, San Diego, CA) and sequenced with Illumina HiSeqX. Read assembly was performed with NOVOPlasty (Dierckxsens et al. 2017) and *Althaea officinalis* (GenBank NC034701) as reference. The consensus chloroplast genome was examined for depth with bowtie2 *via* Geneious Prime (Kearse et al. 2012). Annotation was performed with PLANN (Huang and Cronk 2015) using *Hibiscus rosa-sinensis* MK382984 as reference and the plastome drawn with OGDRAW (Greiner et al. 2019). We used ECuADOR (Carrion et al. 2020) to identify limits between inverted repeat (IR) regions of *M. wigandii* and a set of 10 representative species for phylogenetic analysis and IRs junctions were visualized with IRScope (Amiryousefi et al. 2018). MAFFT version 7 (Katoh and Standley 2013) was used to align sequences and the phylogenetic reconstruction using maximum likelihood (ML) (Figure 1) under a TVM + F + I + G4 model in IQ-TREE1 (Nguyen et al. 2015) run on CIPRES (Miller et al. 2010).

**Figure 1.**
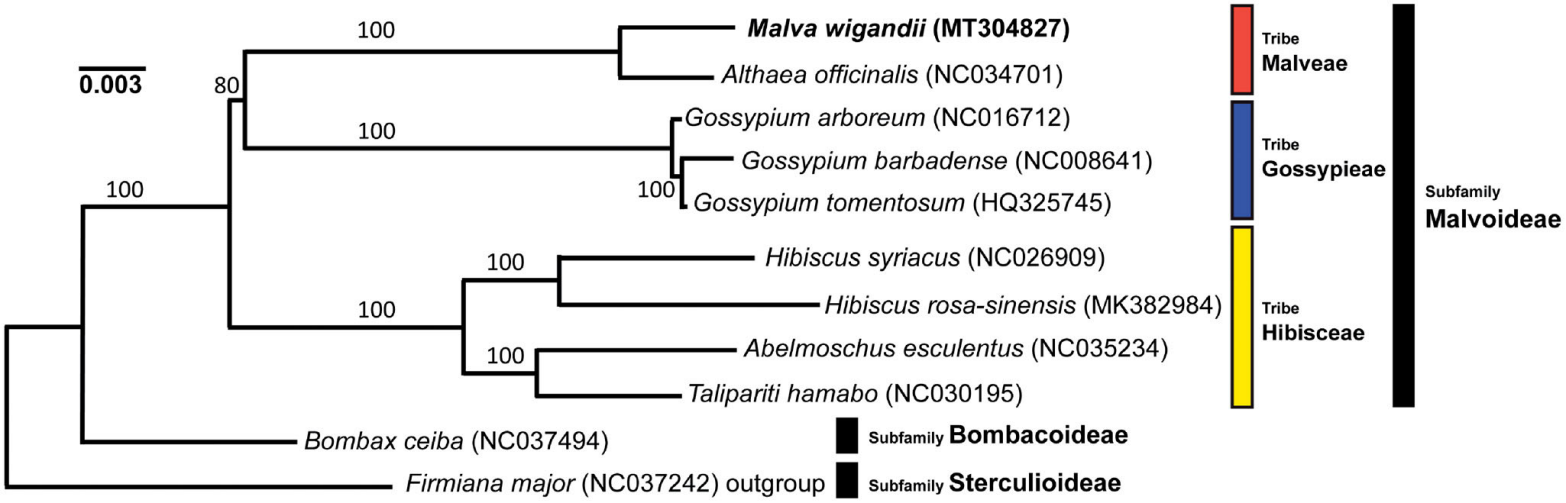
Phylogenetic relationships of *Malva wigandii* with representative species from other tribes in subfamily Malvoideae. The maximum likelihood (ML) phylogeny tree was reconstructed based on 11 chloroplast genome sequences deposited in GenBank. Numbers along branches represent non-parametric bootstrap support.

The plastome of *M. wigandii* has 158,162 bp, exhibiting a typical genome structure common to families in the order Malvales. It possesses a pair of inverted repeat regions (IRa and IRb) of 25,166 bp, a large single copy region of 88,860 bp, and a small single copy region of 20,970 bp. The G + C content of the whole plastome is 37.1%. There are 131 genes, including 85 protein-coding, eight rRNA, and 37 tRNA, seven of which are in the IR. Among the detected genes, 11 have one or two introns. The phylogenetic tree recovers three lineages within the subfamily Malvoideae, corresponding to the three tribes recognized: Malveae, Gossypieae, and Hibisceae, establishing a sister position between Gossypieae and Malveae, in which the genus *Malva* is included as predicted by a nuclear phylogeny (Tate et al. 2005). An interesting trend observed is that while in the late-diverging tribe Malveae the *ycf1* portion included in IRb is just 2–3 bp long, in early diverging tribes Gossypieae and Hibisceae is 79–83 and above 600 bp long, respectively. The genes with the highest number of polymorphic sites for the species examined are *ycf1*, *rpoC2*, *ndhF*, *trnK*, *matK*, and *rpoB.*

## Data Availability

The chloroplast genome sequence of *Malva wigandii* has been deposited in GenBank under MT304827 (https://www.ncbi.nlm.nih.gov/nuccore/MT304827.1/). The data supporting this finding are available in NCBI Bioproject ID PRJNA613244 (https://www.ncbi.nlm.nih.gov/bioproject/?term=PRJNA613244), Biosample ID SAMN17320626 (https://www.ncbi.nlm.nih.gov/biosample/?term=SAMN17320626) and SRA SRX9910562 (https://www.ncbi.nlm.nih.gov/sra/?term=SRX9910562).
